# Current Perspectives on Human Papillomavirus (HPV): Strengthening the Evidence Base for Global Elimination

**DOI:** 10.3390/biomedicines13102374

**Published:** 2025-09-28

**Authors:** Vanja Kaliterna, Tomislav Meštrović

**Affiliations:** 1 Teaching Institute for Public Health of Split and Dalmatia County, 21000 Split, Croatia; 2 Faculty of Health Sciences, University of Split, 21000 Split, Croatia; 3 University Centre Varaždin, University North, 42000 Varaždin, Croatia; 4 Institute for Health Metrics and Evaluation, University of Washington, Seattle, WA 98105, USA

## 1. Introduction

The infection with human papillomavirus (HPV) represents one of the most salient public health challenges on a global level [1], with well-established links to cervical cancer and anogenital malignancies, but also a growing recognition of its role in oropharyngeal cancers [2,3,4,5]. Persistent infection with high-risk HPV types is basically a prerequisite for cervical cancer, making it both a target for prevention and a powerful example of virus-related oncogenesis [2,5]. The availability of effective HPV vaccines and increasingly accessible screening methods has made the elimination of cervical cancer a tangible goal—yet one that remains unevenly pursued across different regions of the world [6,7].

In recent years, the scientific and public health communities have demonstrated renewed interest in HPV-related research [8]. This momentum has been shaped by advances in molecular diagnostics, vaccine technology, implementation science, various treatment possibilities, as well as by the exploration of HPV’s broader health and societal impacts [9,10,11,12,13]. Despite this progress, significant disparities persist in vaccine coverage, access to screening and public awareness, which is especially pertinent for low- and middle-income countries and for marginalized populations in high-income settings [14].

In response to these challenges, the World Health Organization (WHO) launched the Global Strategy to Accelerate the Elimination of Cervical Cancer in 2020, setting ambitious “90–70–90” targets by 2030 for HPV vaccination, screening and treatment [15]. Along those lines, WHO also issued guidelines for primary screening and treatment, which recommend that, for the general population of women, HPV DNA testing should begin at age 30 and be conducted every 5 to 10 years, using either a screen-and-treat or a screen, triage and treat approach [16]. These global commitments have catalysed research, policy development and cross-sectoral collaboration aimed at understanding and overcoming the scientific and social barriers to equitable HPV prevention and control [15,16].

This Editorial pertains to the Special Issue entitled “Current Perspectives on Human Papillomavirus (HPV)” in the journal *Biomedicines*, which brings together a collection of multidisciplinary, original research articles and reviews that reflect the evolving landscape of HPV science and public health [17,18,19,20,21,22]. The contributions to the issue span molecular epidemiology, co-infections, biomarker development, multi-site risk profiling and under-recognized clinical manifestations, offering contemporary insights into the pathobiology, screening/diagnostic strategies and also broader implications of HPV (such as population-level risk factors) [17,18,19,20,21,22]. Together, we believe the content informs and inspires further inquiry into one of the most important infectious agents in global health.

## 2. An Overview of Published Articles

Nipčová Džundová et al. [17] conducted a robust case–control study exploring anal and oral HPV co-infections in women with severe cervical lesions, i.e., cervical intraepithelial neoplasia grade 2 or worse (CIN2+). By analysing 473 women with CIN2+ and 245 controls, they identified the strongest risk factor for anal HPV as the presence of cervical HPV infection. Behavioural factors—such as history of anal intercourse, non-coital anal contact and multiple sexual partners—also significantly increased anal HPV risk. For oral HPV, both self- and partner-reported histories of genital warts emerged as significant predictors. It also illustrates how anatomical site-specific risk factors differ and reinforces the cervix as a key reservoir of high-risk HPV. These insights definitely underscore the necessity of integrated multi-site screening strategies in high-risk populations.

Expanding the diagnostic frontier to non-cervical anatomical sites further, Pauciullo et al. [18] investigated methylation profiles in oral and anal samples from HIV-positive men who have sex with men (MSM), which is a high-risk group for HPV-related cancers. Using a pertinent molecular assay and methylation kits, the study showed significant associations between methylation markers (especially ASCL1 and ZNF582) and lesion severity in anal samples. An important finding was a 30% increase in methylation (ddCt ratio) in high-grade lesions, as well as common multiple HPV infections. This supports the idea of potentially using methylation-based biomarkers in the early detection of HPV-driven malignancies beyond the cervix, particularly in immunocompromised populations.

Along those lines, Mortaki et al. [19] evaluated the clinical performance of DNA methylation markers as a triage tool for high-risk HPV-positive women, both independently and in combination with cervical cytology. In their prospective study on 170 patients, methylation testing alone achieved a sensitivity of 69.7% and specificity of 79% for detecting CIN2+ lesions. The diagnostic accuracy was quantified using the area under the receiver operating characteristic curve (AUC), which reached 0.796 for methylation alone—a value suggesting fair-to-good discriminative ability. Generally, AUC values range from 0.5 (no discrimination) to 1.0 (perfect accuracy), with higher values indicating better test performance across all thresholds. When combined with cytology, diagnostic accuracy improved significantly: sensitivity rose to 94.7% and AUC increased to 0.860, indicating good diagnostic performance. In genotype-stratified analysis, the combination method was particularly effective in HPV16/18-positive women, achieving an AUC of 0.917. Therefore, it is safe to say that the study affirms the clinical utility of methylation markers in risk stratification and provides a strong rationale for incorporating molecular triage into HPV-based screening programs, which can be seen as a step forward in optimizing cervical cancer prevention pathways.

In a rare exploration of the role of HPV in urothelial malignancies, Ahmed et al. [20] conducted an exploratory molecular study on 55 bladder tissue samples from a UK-based population. Their results reveal the presence of high-risk HPV DNA in 33% of malignant bladder samples, predominantly HPV 16, 35 and 52. No benign specimens tested positive. Moreover, immunohistochemistry confirmed HPV E7 protein expression in the majority of HPV DNA-positive cases, suggesting transcriptional activity. Looking at these findings, it is clear that they contribute very important preliminary evidence to the contested role of HPV in bladder carcinogenesis and call for further mechanistic/epidemiological studies to elucidate causality.

Kaliterna et al. [21] presented a large-scale retrospective cross-sectional study of 1211 HPV-positive women in Southern Croatia, providing a detailed genotype-specific landscape of high-risk HPV types and their microbial co-detections. HPV 16 (23.3%) and HPV 31 (22.4%) emerged as the most prevalent genotypes, with HPV 51 and 18 also detected at notable frequencies. The authors investigated age associations and unveiled correlations between high-risk HPV infections and pathogens such as *Chlamydia trachomatis* (*C. trachomatis*), *Gardnerella vaginalis* (*G. vaginalis*) and *Streptococcus agalactiae*. Multinomial logistic regression revealed complex microbial interactions, with *C. trachomatis* negatively associated with 16 related genotypes and *G. vaginalis* positively associated with the same genotypes. This underlines the importance of considering the broader cervico-vaginal microbiome in HPV screening and highlights the need for genotype-tailored regional prevention strategies.

This Special Issue also included a comprehensive narrative review paper by Maghiar et al. [22], focusing on the cutaneous manifestations of HPV infection: from benign lesions such as common warts to malignant skin tumors. By synthesizing evidence from 135 studies published since 2000, the authors outlined the pathophysiological mechanisms of HPV in the skin, identified relevant risk factors, and evaluated diagnostic modalities including biopsy and molecular testing. The review also examined current therapeutic approaches, such as cryotherapy and laser therapy, while noting the limitations and recurrence risks that often accompany these treatments. Importantly, the authors emphasized the preventive potential of HPV vaccination in reducing cutaneous HPV burden and called for greater public education and wider vaccine coverage. The article highlighted the need for innovative therapies and better integration of dermatologic HPV into broader research and policy agendas, positioning skin lesions as an often-overlooked, but nonetheless clinically significant component of HPV-associated diseases.

## 3. Conclusions

The contributions to this Special Issue reflect a very important moment in the global response to HPV—where decades of virological insight are converging with molecular innovation and population health imperatives [10,11,12,13,14]. Rather than focusing solely on established cervical pathways, the collected studies broaden the lens to include multi-site infections, underexplored malignancies and novel diagnostic strategies, pushing the boundaries of how HPV is understood and subsequently addressed in clinical and public health contexts [17,18,19,20,21,22].

The presented work points toward several converging avenues for future research and practice. Across different settings and populations, the need for integrated diagnostic frameworks that combine molecular assays, methylation biomarkers and traditional cytology emerges as a clear priority [18,19,21]. At the same time, the evidence underscores the importance of examining HPV in its broader microbial and behavioral context, where co-infections, sexual practices and immunological status all shape transmission and disease progression [17,21]. Equally, expanding the scope of inquiry beyond cervical disease to include oral, anal, cutaneous, and even urothelial manifestations will be vital in capturing the full spectrum of HPV’s oncogenic potential [17,20,22]. These shared directions reveal the need for an interdisciplinary approach that links virology, microbiology, oncology, behavioural science and public health in pursuit of comprehensive HPV control.

What emerges from these diverse perspectives is a shared recognition: HPV is not a static or narrowly defined threat, but a dynamic and multifaceted challenge that intersects with a myriad of different, aforementioned determinants. This notion arrives at a time of strategic urgency. Global ambitions to eliminate cervical cancer cannot be realized through Pap smears and vaccine utilization alone. They require sustained investment in regionally tailored research, accessible molecular screening, and inclusive prevention strategies that reach beyond the cervix, beyond women, and beyond traditional silos in medicine and science [15,16,23].

Consequently, this Special Issue is not a summation of what we know about HPV—it is rather an invitation to rethink what remains to be discovered, and to pursue that knowledge with the same urgency we bring to prevention. We believe the path forward lies in combining fundamental research with innovation and ensuring that scientific advances translate into tangible health equity on a global scale.
